# Supplementary material for: Antibiotic Prescribing Decisions for Upper Respiratory Tract Infections Among Primary Healthcare Physicians in China: A Mixed-Methods Approach Based on the Theory of Planned Behavior
Source: Antibiotics (Basel). 2024 Nov 20;13(11):1104. doi: 10.3390/antibiotics13111104 (PMC11591080; doi:10.3390/antibiotics13111104)
Supplement: Supplementary file 1 [file antibiotics-13-01104-s001.zip › antibiotics-3270943-supplementary File S1.pdf]

Table S1 Gross Domestic Product (GDP) ranking of each prefecture-level region of Shaanxi Province in 2020

Prefecture-level cities	GDP (billions Chinese Yuan)	Ranking of GDP
Xi'an	932.119	1
Yulin	413.628	2
Baoji	222.725	3
Xianyang	219.533	4
Weinan	182.847	5
Yan'an	166.389	6
Hanzhong	154.759	7
Ankang	118.206	8
Shangluo	83.721	9
Tongchuan	35.472	10

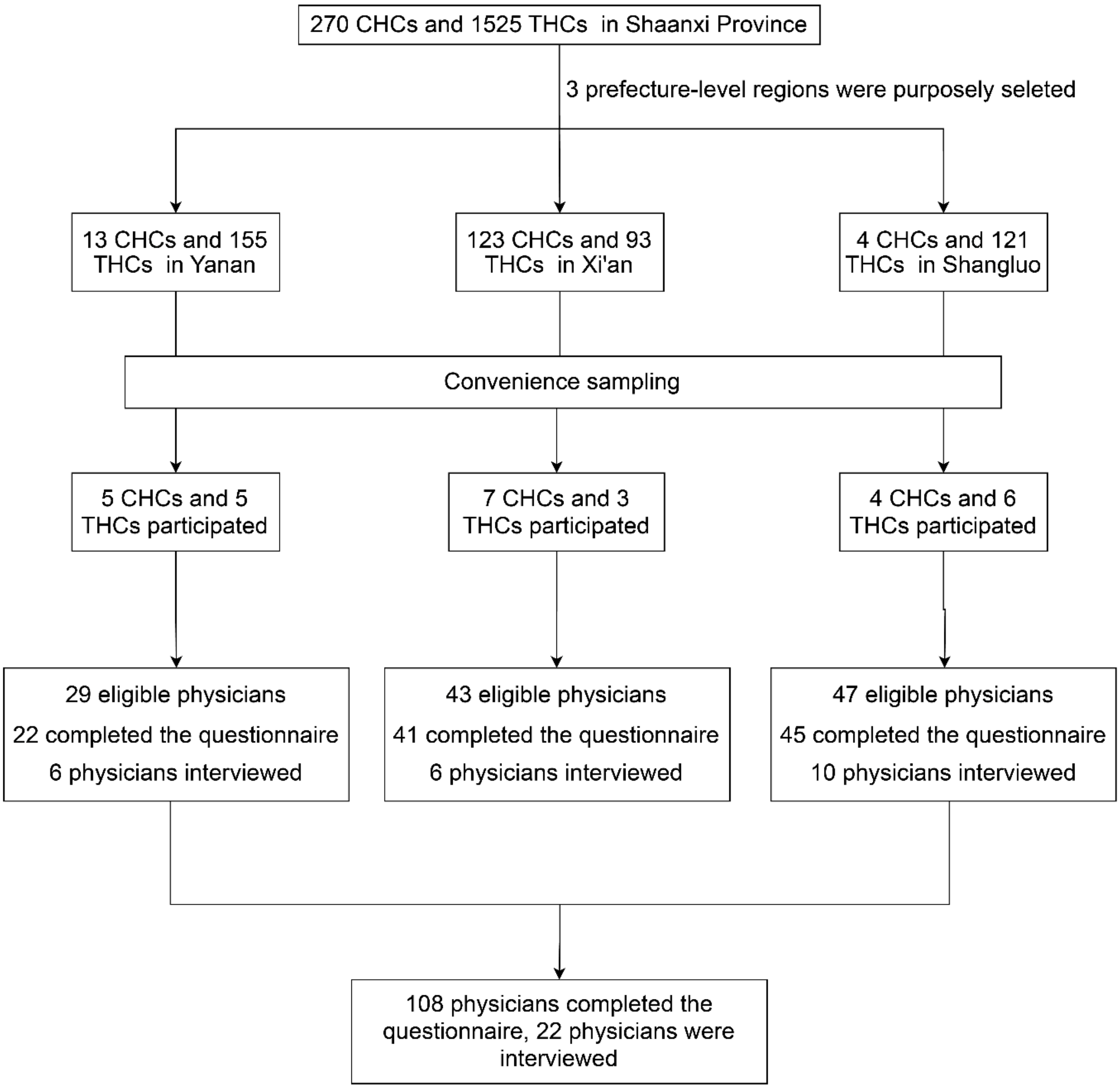

Figure S1 Participant recruiting procedures

Note: CHCs: Community healthcare centers. THCs: Township healthcare centers

Table S2 Survey responses

Items	Strongly agree	Agree	Neural	Disagree	Strongly disagree
**Attitudes**					
**Awareness of AMR**					
AMR is a major public health issue in China	79	25	3	1	0
Antibiotic over-prescription leads to AMR	95	13	0	0	0
Antibiotic over-prescription is common in China's PHC facilities	39	33	27	6	3
**Beliefs in antibiotics**					
Antibiotics can relieve symptoms of URTIs	3	4	30	45	26
Antibiotics can lower the relapse rate of URTIs	3	9	30	29	37
Antibiotics can prevent the exacerbation of URTI symptoms	3	6	53	32	14
Antibiotics can prevent severe complications from URTIs	2	10	38	40	18
**Beliefs in prior prescribing experience**					
Prior experience is important if diagnostic tests are unavailable	8	62	24	11	3
Prior experience is important in PHC physicians' daily practice	15	47	31	10	5
Prior experience can improve consultation efficiency	8	38	41	15	6
Most of my clinical decisions are made according to prior clinical experience	4	36	41	15	12
Prior experience is important when facing diagnostic uncertainty	10	43	30	19	6
**Subjective norms**					
Senior physicians' antibiotic prescribing behavior can affect me	13	42	33	15	5
Peer physicians' antibiotic prescribing behavior can affect me	6	22	45	24	11
Patients always want me to prescribe antibiotics	14	55	26	10	3
Parents always want me to prescribe antibiotics for their kids	10	55	28	12	3
**Perceived behavior control**					
The decision to prescribe an antibiotic is not entirely up to me	8	9	22	37	32
I prescribe antibiotics when patients strongly ask for them	2	5	29	48	24

I prescribe antibiotics when parents strongly ask for them	1	4	39	25	39
I prescribe antibiotics when I do not have time to assess patients' conditions	0	4	19	43	42
I prescribe antibiotics when I do not have time to explain my decision to patients	1	1	10	31	65
**Behavior intentions**	Never	Seldom	Sometimes	Often	Always
For URTI patients with following symptoms, the degree to which you are willing to prescribe antibiotics:					
Purulent sputum	7	9	28	23	41
Nasal discharge	15	22	21	26	24
Sore throat	27	27	33	14	7
Fever over 38.5℃	19	22	28	23	16
Suppurative tonsillitis	0	6	8	28	66
Cough	6	21	24	22	35
