# Supplementary material for: Antibiotic Prescribing Decisions for Upper Respiratory Tract Infections Among Primary Healthcare Physicians in China: A Mixed-Methods Approach Based on the Theory of Planned Behavior
Source: Antibiotics (Basel). 2024 Nov 20;13(11):1104. doi: 10.3390/antibiotics13111104 (PMC11591080; doi:10.3390/antibiotics13111104)
Supplement: Supplementary file 1 [file antibiotics-13-01104-s001.zip › antibiotics-3270943-supplementary File S2. Inform Consent.pdf]

Antibiotic prescribing for acute upper respiratory tract infections in primary healthcare facilities in Shaanxi Province, China.

Interview Guide

Information sheet

Introduction: I am going to give you information and invite you to be part of this research. You do not have to decide today whether or not you will participate in the research. Before you decide, you can talk to anyone you feel comfortable with about the research.

This information sheet may contain words that you do not understand. Please ask me to stop as we go through the information and I will take time to explain.

Purpose: Antimicrobial resistance is growing throughout the world. The aim of the current study is to get an insight of antibiotic prescribing behaviour among primary healthcare physicians in Shaanxi Province, China. The information you provide will help us improve our understanding of practice of antibiotic prescription and our intervention refinement.

Participant Selection: You are being invited to take part in this research because we feel that your experience as a primary healthcare physician can contribute much to our understanding and knowledge of antibiotic prescription in primary healthcare settings. Your participation in this research is entirely voluntary. It is your choice whether to participate or not.

Methodology: Qualitative study design based on semi-structured interview method will be used. Our conversation will be audio recorded and analyzed by a research team.

Confidentiality: We will not be sharing information about you to anyone outside of the research team. The information that we collect from this research project will be kept private. All information is confidential. No name or personal information will be mentioned when the research is published.

Right to Refuse or Withdraw: You do not have to take part in this research if you do not wish to do so, and you may stop participating in the interview at any time that you wish.

Consent Form

I have read the foregoing information, or it has been read to me. I have had the opportunity to ask questions about it and any questions I have been asked have been answered to my satisfaction. I consent voluntarily to be a participant in this study.

Name: ____________________ Date: ____________________ Signature: _________________

Topic guide

Part I: Demographic information

1. Facility type
2. Gender
3. Age
4. Highest education level
5. Working years
6. Professional title
7. How many training sessions on antibiotics you participate in the last twelve months?

Part II: Research questions

1. What do you know about antimicrobial resistance (AMR)?

   Probes:

   - What do you think of the current trend of AMR in China?
   - What do you know about the causes and consequences of AMR?
   - To what extent are primary healthcare physicians responsible for AMR? Why?
2. Could you please describe the common diseases in your facilities?

   Probe:

   - How often do you come across patients with acute URTIs?
3. What is your treatment for acute URTI cases?

   Probes:

   - Could you please describe the circumstances in which you prescribe antibiotics for acute URTI patients?
   - What are the commonly prescribed antibiotics? Why do you prescribe them?
   - How confident do you feel in your prescribing decisions?
   - What will you do if you are uncertain about the diagnosis?
4. What are your thoughts on the use of antibiotics for URTIs?

   Probes:

   - How necessary do you think antibiotics are in treating URTIs?

- Do you believe there are any potential harms in not prescribing antibiotics to patients with URTIs? Why?

5. What are the factors that influence your antibiotic prescription?

   Probes:

   - What factors are particularly important and why?
   - Do any external pressures or constraints affect your decisions? Why?

6. What resources or factors increase your confidence in making appropriate prescribing decisions for URTIs? For example, guidelines, diagnostic tests, training, etc. What do you think about them?
7. Is there anything you'd like to add about prescribing antibiotics that is important in your opinion?
